# Regulation of p73 by Hck through kinase-dependent and independent mechanisms

**DOI:** 10.1186/1471-2199-8-45

**Published:** 2007-05-30

**Authors:** Preeti Paliwal, Vegesna Radha, Ghanshyam Swarup

**Affiliations:** 1 Centre for Cellular and Molecular Biology, Uppal Road, Hyderabad-500007, India

## Abstract

**Background:**

p73, a p53 family member is a transcription factor that plays a role in cell cycle, differentiation and apoptosis. p73 is regulated through post translational modifications and protein interactions. c-Abl is the only known tyrosine kinase that phosphorylates and activates p73. Here we have analyzed the role of Src family kinases, which are involved in diverse signaling pathways, in regulating p73.

**Results:**

Exogenously expressed as well as cellular Hck and p73 interact *in vivo*. *In vitro *binding assays show that SH3 domain of Hck interacts with p73. Co-expression of p73 with Hck or c-Src in mammalian cells resulted in tyrosine phosphorylation of p73. Using site directed mutational analysis, we determined that Tyr-28 was the major site of phosphorylation by Hck and c-Src, unlike c-Abl which phosphorylates Tyr-99. In a kinase dependent manner, Hck co-expression resulted in stabilization of p73 protein in the cytoplasm. Activation of Hck in HL-60 cells resulted in tyrosine phosphorylation of endogenous p73. Both exogenous and endogenous Hck localize to the nuclear as well as cytoplasmic compartment, just as does p73. Ectopically expressed Hck repressed the transcriptional activity of p73 as determined by promoter assays and semi-quantitative RT-PCR analysis of the p73 target, Ipaf and MDM2. SH3 domain- dependent function of Hck was required for its effect on p73 activity, which was also reflected in its ability to inhibit p73-mediated apoptosis. We also show that Hck interacts with Yes associated protein (YAP), a transcriptional co-activator of p73, and shRNA mediated knockdown of YAP protein reduces p73 induced Ipaf promoter activation.

**Conclusion:**

We have identified p73 as a novel substrate and interacting partner of Hck and show that it regulates p73 through mechanisms that are dependent on either catalytic activity or protein interaction domains. Hck-SH3 domain-mediated interactions play an important role in the inhibition of p73-dependent transcriptional activation of a target gene, Ipaf, as well as apoptosis.

## Background

p73 is a transcription factor that shares significant homology with the tumor suppressor protein p53 and mediates cellular functions such as cell cycle arrest, differentiation, senescence and apoptosis [1,2]. Alternate promoter usage and splicing give rise to p73 variants differing at the N and C-terminus, p73 α and β forms being the predominant transactivation competent forms [3,4]. p73 and p53 bind common response elements and transactivate an overlapping set of target genes, though with differing efficiencies [1,5-7]. p73 knockout mice exhibit severe neurological, inflammatory and pheromonal defects but lack spontaneous tumor formation [8]. Forced expression of p73 activates target genes like p21, Bax, IGF BP3, CyclinG, Mdm2, caspase-1, etc and promotes apoptosis in both p53 positive as well as negative cell lines [1,5-7,9].

Cellular p73 protein is maintained at very low levels and activation is controlled at transcriptional as well as post-translational levels. p73 is induced in response to a range of DNA damaging agents as well as during differentiation of many cell types [10]. Multiple mechanisms like post-translational modifications, protein-protein interaction and sub-cellular compartmentalization regulate transactivation potential and apoptosis inducing property of p73 [10,11]. Several viral and cellular proteins interact with p73 and regulate its activity [3]. While some of the interacting proteins enhance transactivation potential of p73 by stabilizing it [12-14], others inhibit transactivation property of p73 [15-20]. p73 is also known to interact with and cooperate with other transcription factors to regulate gene expression [9,21]. Interaction with c-Abl through SH3 domain in response to DNA damage results in phosphorylation at Tyr99, stabilization and activation [12,22,23]. c-Abl can also activate p38 MAP kinase to phosphorylate p73 leading to its stabilization [24].

Src family kinases (SFKs) are non receptor tyrosine kinases involved in the regulation of diverse cellular functions like proliferation, differentiation, survival, adhesion, motility and angiogenesis [25]. Apart from a catalytic domain, they possess SH2, SH3 and SH4 domains required for sub-cellular targeting and protein interaction and therefore have cellular functions dependent on catalytic activity as well as protein interaction domains [26,27]. Hck is a SFK showing restricted expression in hematopoietic cells of myeloid and monocytic lineage and in embryonic stem cells [28-30]. It exists as two isoforms (p61 and p59 in humans), which arise due to alternate translational start sites [31]. Hck is activated in response to cytokines and is an important component of signaling pathways in activated macrophages [32-34]. Hck levels increase during differentiation of monocytes and it plays a role in phagocytosis, adhesion, respiratory burst, granule secretion and apoptosis [35-37]. Several proteins like Cbl, Stat-3, WASP, WIP, ELMO, ACK1 and C3G interact with Hck through its SH3 domain [36-40]

The mechanisms that regulate stability and pro-apoptotic activity of p73 are distinct from those used by p53 and regulation by tyrosine phosphorylation has been shown only for p73 [23]. Though initial studies described Tyr-99 as the major site of phosphorylation by cAbl, it has been shown that several other tyrosine residues are also targeted [41]. But thus far, no other tyrosine kinase has been described to phosphorylate or regulate p73 activity. Hck interacts with c-Abl and they modulate each other's activity [42]. Given the role of SFKs in mediating survival and apoptotic pathways in cells, we investigated the role of Hck in regulating p73 activity and function. Our results identify p73 as a novel interacting partner and substrate of Hck, and that Hck regulates p73 through kinase dependent as well as independent functions. We also provide evidence for functional interaction between p73 and Hck leading to regulation of p73 induced apoptosis.

## Results

### Hck interacts with p73α

**Figure 1:**
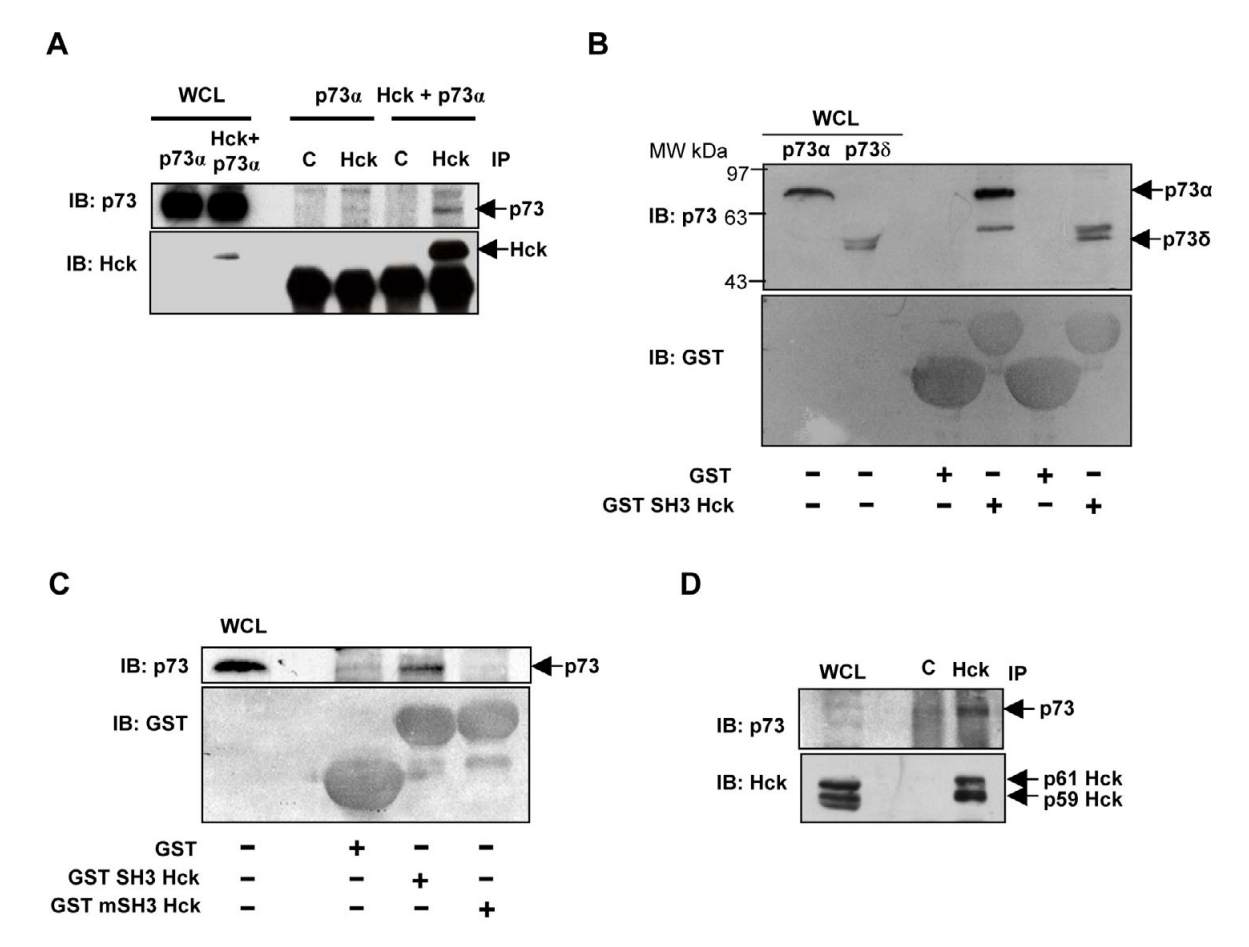
**Hck interacts with p73α *in vitro *and *in vivo***. **(A) **Cos1 cells transiently expressing HA-p73α alone or with Hck were immunoprecipitated with control rabbit IgG (C) or Hck polyclonal antibody. The immunoprecipitates were resolved by SDS-PAGE and subjected to western blotting to detect p73 and Hck. **(B) **GST fusion proteins bound to Glutathione-Sepharose beads were incubated with lysates of Cos1 cells overexpressing p73α and p73δ. Bound proteins were subjected to western blotting to detect p73. **(C) **GST-SH3 Hck and GST-mSH3 Hck recombinant fusion proteins bound to Glutathione-Sepharose beads were incubated with lysates of Cos1 cells overexpressing p73α and bound proteins were subjected to western blotting with p73 antibody. **(D) **Lysates of differentiated HL-60 cells (DMSO 1.25%) were immunoprecipitated with control rabbit IgG or Hck antibodies and subjected to western blotting for p73 and Hck. (WCL indicates whole cell lysate).

We explored the possibility of p73 interacting with Hck *in vivo *in co-immunoprecipitation experiments. p73 was detected in Hck immunoprecipitates from cells expressing both Hck and p73, but not in cells expressing p73 alone suggesting their interaction in mammalian cells (Fig. 1A). p73 has polyproline sequences that could serve as interacting motifs for SH3 domain containing proteins. To determine whether Hck and p73 form a complex through SH3-polyproline mediated interaction, *in vitro *binding experiments were carried out using glutathione-S-transferase (GST) fusion proteins. Recombinant fusion protein of GST with SH3 domain of Hck (GST-SH3 Hck) bound to Glutathione Sepharose beads was incubated with lysates of Cos1 cells transfected with p73α and p73δ. p73δ is the shortest isoform which lacks the entire C-terminus including the SAM domain[3]. p73α and p73δ bound to GST-SH3-Hck and not with GST alone (Fig. 1B). Further, recombinant fusion protein of SH3 domain mutant (W93A) disabled for interaction (GST-mSH3 Hck) and GST-SH3-Hck were incubated with lysates of Cos1 cells transfected with p73α. p73α bound to GST-SH3-Hck and not to GST or GST-mSH3-Hck (whose levels were comparable) (Fig. 1C). It has been shown that upon dimethyl sulfoxide (DMSO) treatment of HL-60 cells, both mRNA and protein levels of p73α increase in a time dependent manner [43]. Hck levels are also known to increase under these conditions [44]. To examine whether endogenous p73 interacts with Hck under these conditions, we subjected lysates of differentiated HL-60 cells to immunoprecipitation using Hck antibody. Western blotting with p73 antibody showed that p73α was co-immunoprecipitated with Hck and not with control antibody (Fig. 1D). Our results suggest that endogenous Hck and p73 interact under physiological conditions.

### Hck co-expression results in phosphorylation of p73

**Figure 2:**
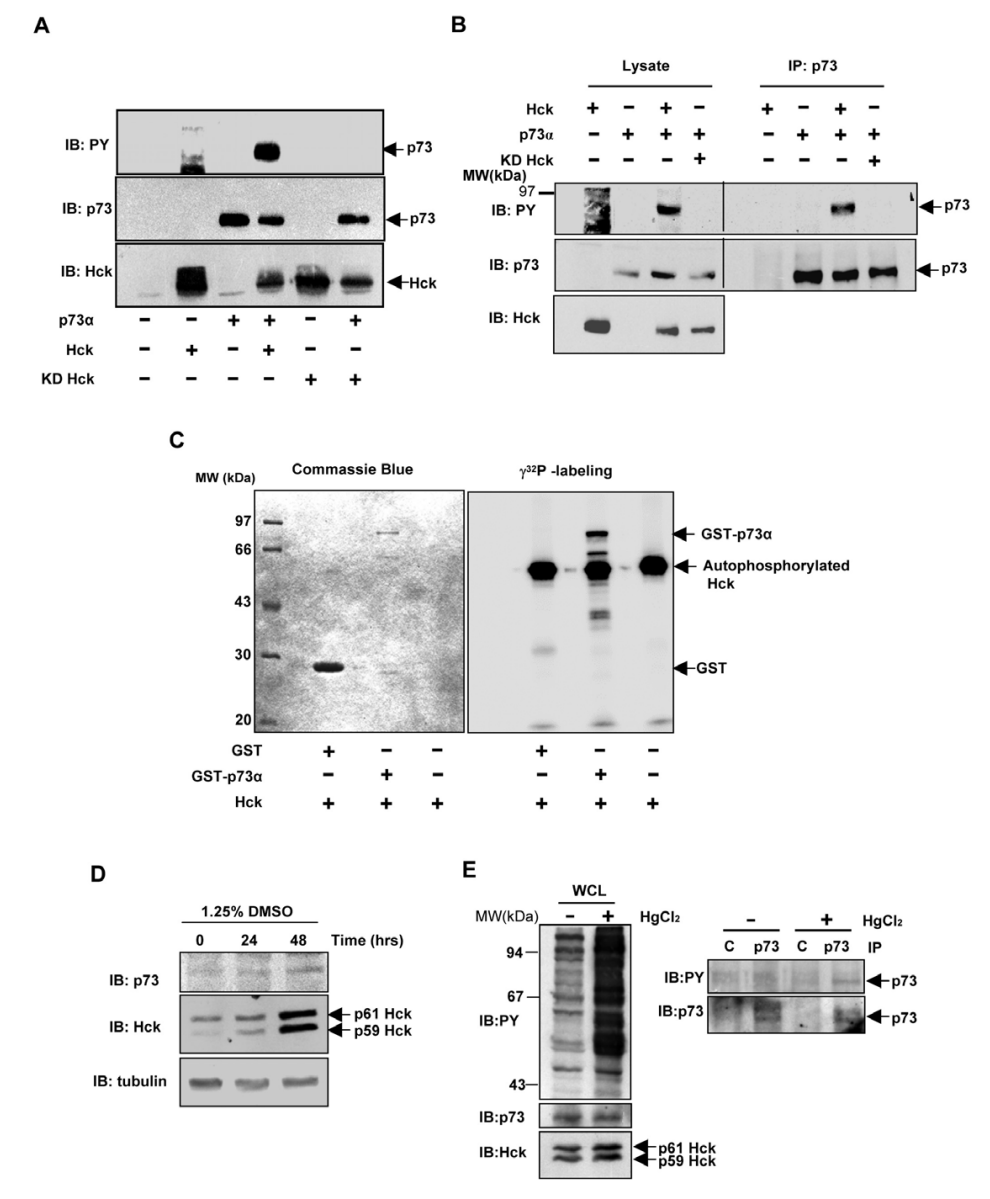
**Hck phosphorylates p73α *in vivo *and *in vitro***. **(A) **Whole cell lysates prepared from Cos1 cells transfected with HA-p73α (1.6 μg) with and without Hck (0.4 μg) expression constructs in the ratio of 4:1 were subjected to immunoblotting with pTyr, p73 and Hck antibodies. **(B) **Cos1 cells transfected with indicated expression constructs were subjected to immunoprecipitation with p73 antibody and immunoprecipitates analyzed for pTyr, p73 and Hck by western blotting. The amount of DNA was kept constant by addition of control vector pcDNA3. **(C) **Purified Hck protein (80 nM) was incubated with GST, GST-p73α and alone with γ^32^P-ATP for 30 minutes at 37°C in an *in vitro *kinase assay. The proteins were analyzed by SDS-PAGE and stained with commassie blue (left panel). The gel was then dried and phosphorylated proteins visualized by phosphor imaging (right panel). **(D) **Western blot showing the endogenous protein levels of p73 and Hck upon differentiation with DMSO in HL-60 cells. Tubulin expression was determined as a loading control. **(E) **Endogenous p73 gets phosphorylated on tyrosine upon activation of Hck. Western blot showing the phosphotyrosine content of cellular proteins and levels of endogenous p73 and Hck upon HgCl_2 _treatment in differentiated HL-60 cells (left panel). Lysates of diferentiated HL-60 cells treated with or without HgCl_2 _were subjected to immunoprecipitation with control (rabbit IgG) or p73 (rabbit polyclonal) antibody and western blotting performed with anti-phosphotyrosine antibody (right panel, upper portion) Immunoprecipitated p73 is shown in right panel, lower portion.

Since p73 interacted with Hck, the possibility of p73 being a substrate of Hck *in vivo *was explored. Cos1 cells were transfected with HA-p73α expression construct with and without Hck and whole cell lysates subjected to western blotting with anti-phosphotyrosine antibody. When p73 was co-expressed with Hck, phosphotyrosine was seen on the polypeptide corresponding to p73 in mobility as detected by reprobing the blot using p73 antibody (Fig. 2A). Cells transfected with p73 or Hck alone did not show any corresponding phosphotyrosine containing peptides. Phosphorylation of p73 was dependent on kinase activity of Hck since co-expression of a catalytically inactive mutant KD-Hck (K269E) did not show tyrosine-phosphorylated polypeptide at the corresponding position (Fig. 2A). To confirm phosphorylation of p73 more directly, p73 was immunoprecipitated from cells expressing p73α and Hck. Phosphorylated p73 could be detected in immunoprecipitates of p73α with Hck over-expression whereas it was not observed with over-expressed KD-Hck (Fig. 2B, upper panel). Hck also phosphorylated p73β, dependent on its kinase activity (data not shown). These results suggested that Hck expression could induce tyrosine phosphorylation of p73α and β *in vivo*. To check whether Hck phosphorylation on p73 is a direct event, *in vitro *phosphorylation assay was carried out. Recombinant GST and GST-p73α bound to glutathione-Sepharose beads were incubated with purified Hck enzyme and γ^32^P ATP. Proteins were analyzed by SDS-PAGE, stained and the gel dried for phosphor imaging. Hck phosphorylated GST-p73α and not GST alone (Fig. 2C, right panel). Protein expression was visualized by commassie staining of the gel prior to exposure (Fig. 2C, left panel). This result showed that Hck could directly phosphorylate p73α.

### Endogenous p73 gets tyrosine phosphorylated upon activation of Hck

Treatment of myelomonocytic cell lines with mercuric chloride (HgCl_2_) has been shown to specifically activate Hck, and has been used to identify substrates of Hck [36,45,46]. Treatment of HL-60 cells with DMSO showed an increase in protein levels of both p73α and Hck (Fig. 2D). To find out whether endogenous p73 gets tyrosine phosphorylated upon activation of Hck, HL-60 cells were differentiated for 48 hours with DMSO and then subjected to HgCl_2 _treatment. The lysates were immunoprecipitated with p73 and control antibody. It was observed that endogenous p73 gets tyrosine phosphorylated upon activation of Hck as determined by western blotting with phosphotyrosine antibody (Fig. 2E, right panel). The left panel of Fig. 2E shows the western blots of whole cell lysate (WCL) with phosphotyrosine, p73 and Hck antibodies.

### Hck phosphorylates p73α predominantly on Tyr28

**Figure 3:**
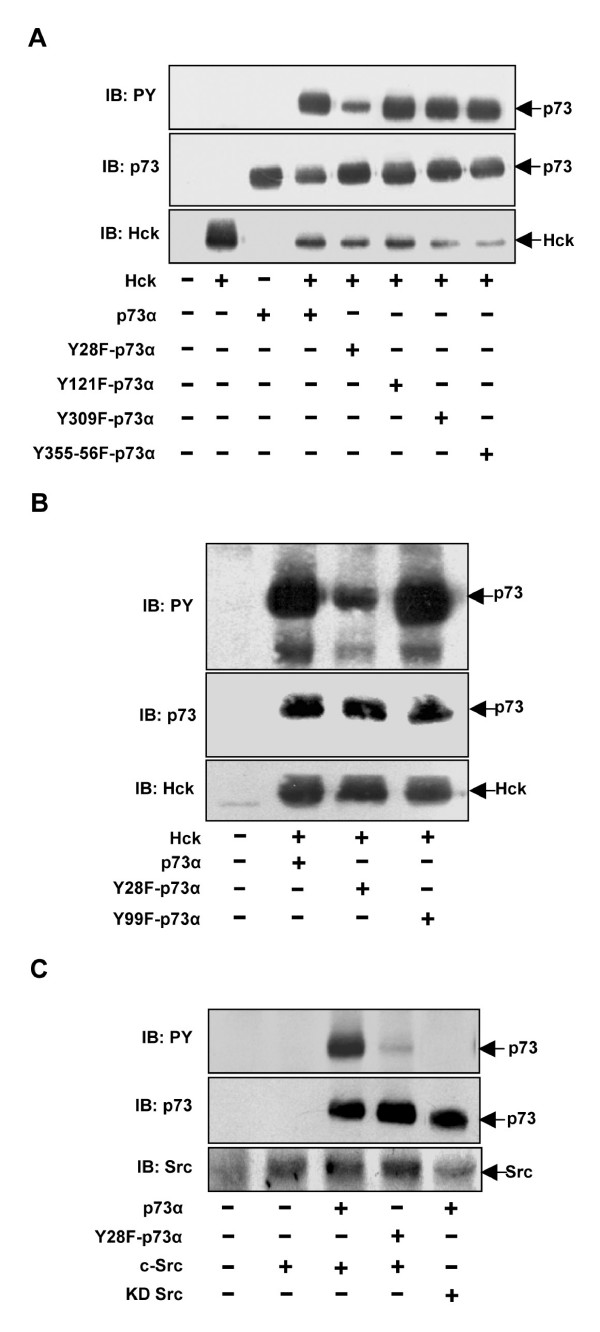
**Tyr-28 is the major site of phosphorylation on p73 upon Hck co-expression**. **(A, B) **Cos1 cells transfected with indicated combinations of p73 and Hck plasmids were analyzed by western blotting for pTyr. The same blot was reprobed for p73 and Hck. **(C) **Src tyrosine kinase phosphorylates p73α on Tyr-28 residue. Cos1 cells transfected with p73α, Y28F-p73α or c-Src and KD-Src were subjected to western blotting for pTyr, p73 and Src.

To identify the site of phosphorylation on p73α by Hck, different tyrosine mutants were constructed by PCR based site directed mutagenesis in which tyrosine residues that are predicted targets for Src family tyrosine kinases (SFKs) were mutated to phenylalanine. Cos1 cells were transfected with p73α or Y28F-p73α, Y121F-p73α, Y309F-p73α, Y355-356F-p73α in the presence of Hck and whole cell lysates subjected to western blotting with pTyr, p73 and Hck antibodies. All the mutants except Y28F-p73α showed phosphorylation comparable to that seen on WT-p73α (Fig. 3A). Y28F-p73α was compromised considerably in phosphorylation by Hck (Fig. 3A). This was not due to lower level of expression because p73 and Hck were comparable as determined by reprobing the blot with anti-p73 and anti-Hck antibody (Fig. 3A). c-Abl is a non-receptor tyrosine kinase which phosphorylates p73α on Y99 residue and activates it [23]. Hck induced phosphorylation of Y99F-p73α as well as WT-p73α while Y28F-p73α was particularly compromised in phosphorylation (Fig. 3B). c-Src co-expression also resulted in p73α phosphorylation predominantly on Y28 residue whereas expression of KD Src did not result in p73 phosphorylation (Fig. 3C). Taken together, our results suggest a novel site (Y28) of phosphorylation on p73α by Src family kinases, which is different from c-Abl mediated phosphorylation of p73 on Y99 residue.

### Hck co-expression stabilizes p73

**Figure 4:**
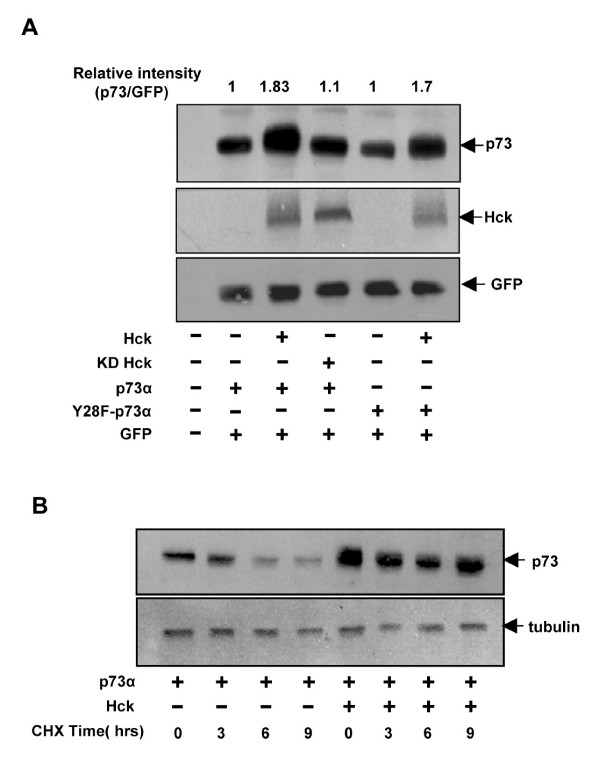
**Hck stabilizes p73α dependent on its kinase activity**. **(A) **Hela cells transfected with GFP (50 ng) and Hck or KDHck(200 ng) in the presence of p73α or Y28F-p73α (50 ng) were subjected to western blotting for p73, Hck and GFP. GFP was used as a transfection efficiency control. **(B) **Hela cells transfected with p73α or p73α and Hck together were treated with cycloheximide (50 μg/ml), harvested at the indicated time periods and immunoblotted for p73 and tubulin. The amount of DNA in transfections was kept constant by the addition of control vector pcDNA3.

To determine whether Hck affects p73 protein levels, Hela cells were transfected with p73α alone or with Hck and whole cell lysates subjected to immunoblotting with anti p73 antibody. Co-expression of Hck but not KD-Hck showed higher p73α protein levels (Fig. 4A). Hck also increased the protein levels of Y28F-p73α, which suggested that increase in p73α protein level is not dependent on Y28 phosphorylation. The same blot was probed for Hck and GFP that was used as transfection efficiency control (Fig. 4A). These results suggest that Hck could increase the p73 protein levels in a kinase dependent manner. To examine whether p73 increase seen upon co-expression with Hck is due to effect on its stability, levels of p73 protein were determined in the presence of cycloheximide. p73 levels dropped significantly by 9 hours of cycloheximide treatment but did not change significantly when co-expressed with Hck (Fig. 4B) suggesting that Hck increases p73 protein stability.

### Sub-cellular localization of Hck and p73

**Figure 5:**
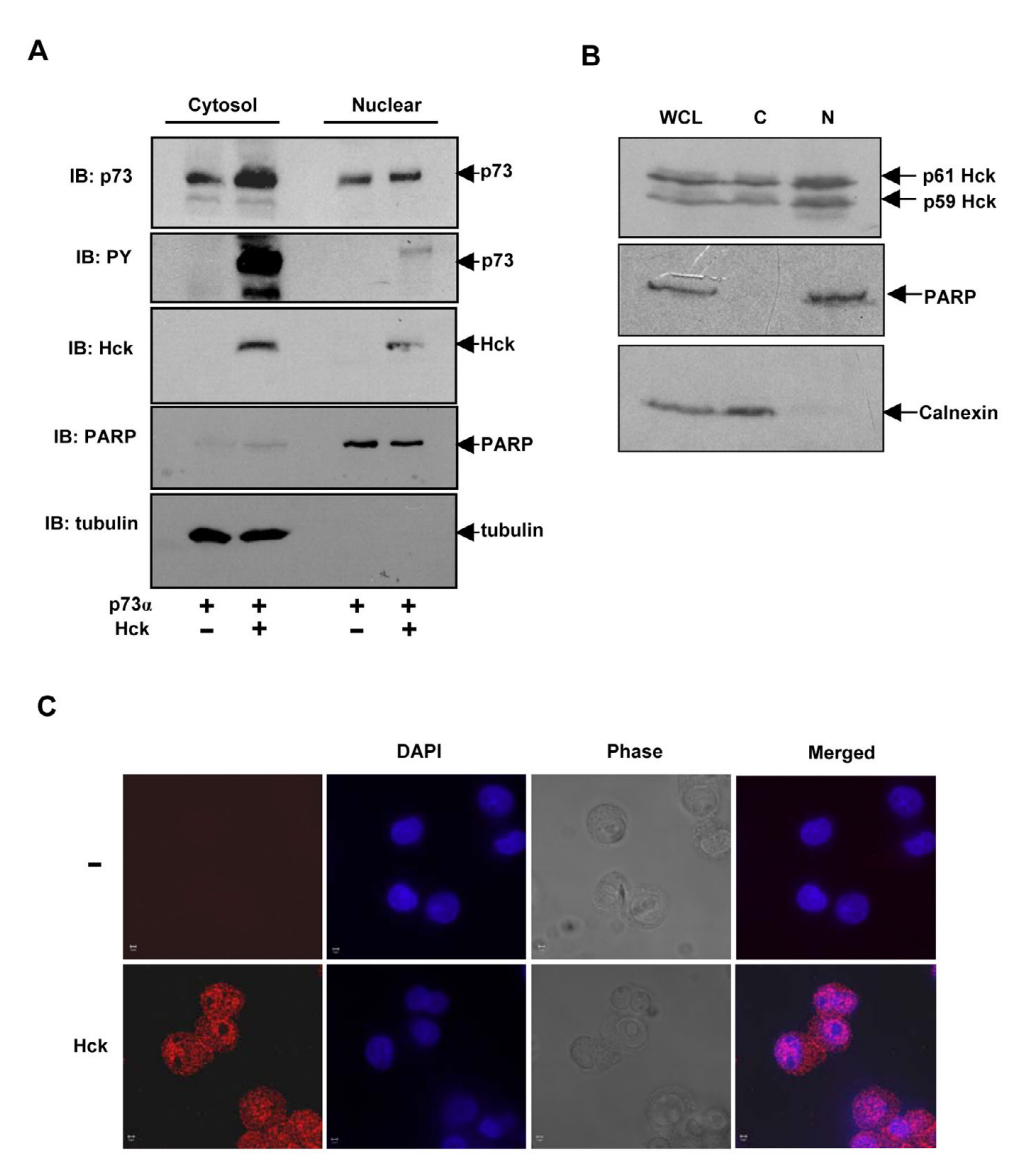
**Sub-cellular localization of Hck and p73α**. **(A) **Cos1 cells transiently transfected with p73α or Hck and p73α were fractionated into nuclear and cytosolic fractions and analysed by immunoblotting for pTyr, p73 and Hck. The purity of nuclear and cytosolic fractions was confirmed by immunoblotting for PARP and α-tubulin. **(B) **Endogenous Hck in HL-60 cells localizes to both cytosolic and nuclear fractions. HL-60 cells were fractionated into nuclear and cytosolic (post nuclear fraction) fractions and immunoblotted using Hck antibody. The purity of fractions was confirmed by immunoblotting with PARP and Calnexin. WCL indicates whole cell lysates. **(C) **Localization of endogenous Hck in the nucleus by immunostaining. HL-60 cells were immunostained with Hck antibody after 24 hours of differentiation by TPA (10 ng/ml) and analysed by confocal microscopy. The image shown is the central section passing through nucleus. The cells were also stained without primary antibody, which served as a control.

Since Hck is a non-receptor tyrosine kinase known to localize to plasma membranes, Golgi and in the cytoplasm [47-50] and p73 shows nucleo-cytoplasmic exchange, we investigated sub-cellular distribution of p73α upon co-expression with Hck by biochemical fractionation of transfected Cos1 cells. Phosphorylated p73α was present predominantly in cytosolic fraction upon Hck over expression (Fig. 5A). When co-expressed with Hck, there was 2.3-fold increase in cytosolic p73 as compared to the cytosolic fraction of p73α transfected alone (Fig. 5A) suggesting that p73 is stabilized in the cytosolic fraction. Surprisingly, we observed that a fraction of Hck localized to the nucleus. Cross-contamination of the fractions was ruled out by probing for tubulin and PARP.

Unlike other Src family kinases such as Src, Lyn and Fyn [51-55], no evidence is available for nuclear localization of Hck. It was therefore important to evaluate that presence of Hck in the nucleus is not a feature of exogenously expressed protein. We determined the sub-cellular distribution of endogenous Hck in a myeloid cell line HL-60. As shown in Fig. 5B, endogenous Hck distributed to both nuclear and cytosolic compartments as evidenced by sub-cellular fractionation. To confirm that sub cellular fractions were not contaminated, the fractions were analysed by western blotting for Calnexin (an ER protein, present in post nuclear fraction) and PARP (present only in nuclear fraction). The presence of endogenous Hck in the nucleus was confirmed by indirect immunofluorescence assay. HL-60 cells were differentiated with 10 ng/ml of 12-*O*-tetradecanoylphorbol-13-acetate (TPA), which allows cells to adhere to the coverslips. After 24 hours, cells were fixed and immunostained with Hck antibody and analysed by confocal microscopy. The middle section passing through the nucleus was analysed for determination of Hck localization. As shown in Fig. 5C, endogenous Hck localized to the nucleus. The cells were also stained without primary antibody for control.

### Hck suppresses transactivation of p73 targets

**Figure 6:**
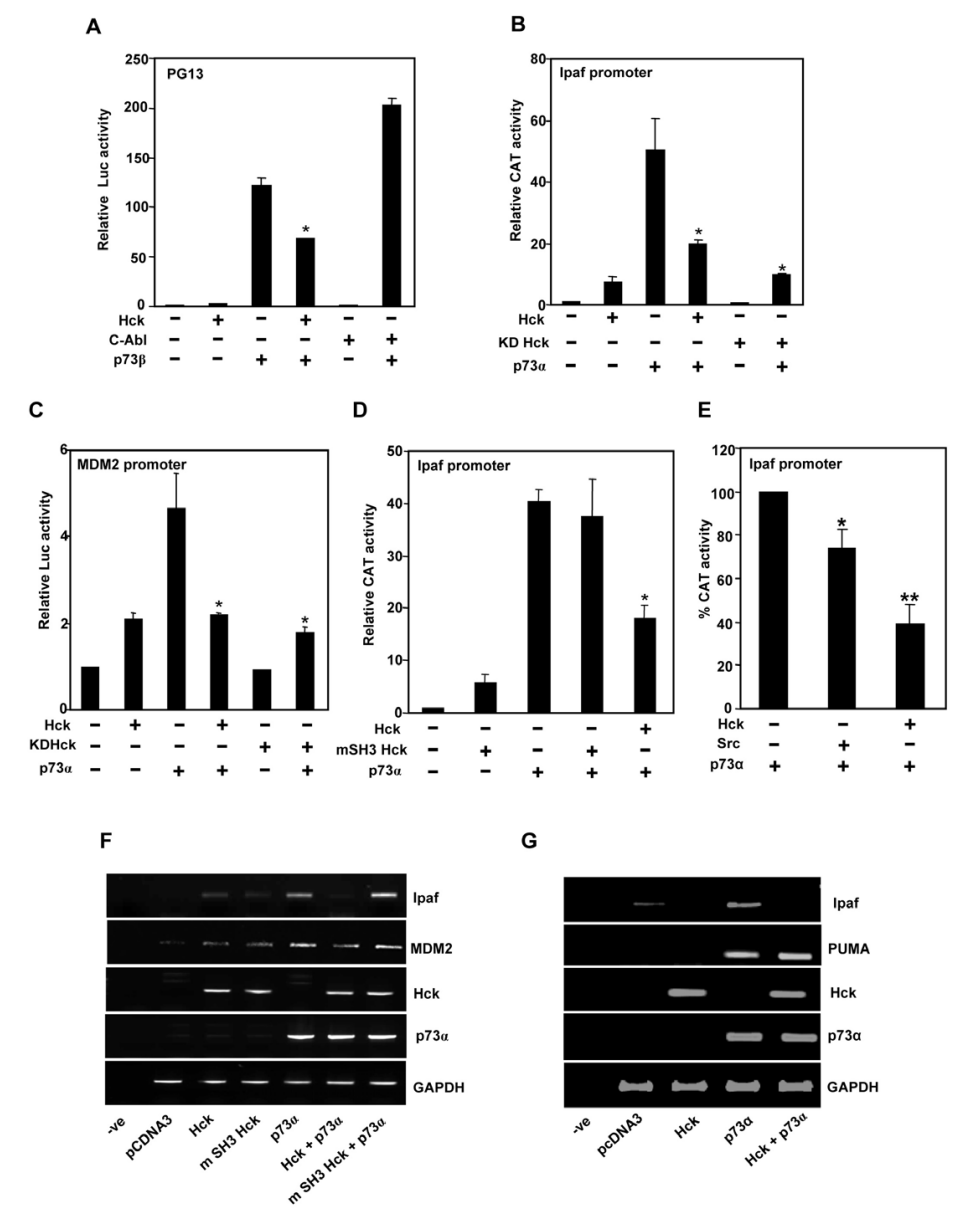
**Hck inhibits the transcriptional activity of p73α and β isoforms, dependent on its SH3 domain**. **(A-D) **Hela cells transfected with pCMV-βgal (50 ng) and different promoter constructs (100 ng) along with p73α or p73β and Hck or KD-Hck were subjected to either luciferase or CAT activity measurements. The amount of DNA was kept constant in all transfections to 400 ng by adding pcDNA3. Hck, KD-Hck, mSH3-Hck and c-Abl plasmids were used at 100 ng for different promoter constructs. The amount of p73α used was 2 ng for Ipaf-CAT promoter and 50 ng for MDM2-Luc promoter. The amount of p73β used was 10 ng for PG13-Luc promoter. Relative reporter activities were calculated after normalizing with β-galactosidase activities. Data presented here are mean ± S.D. of at least three independent experiments, *p < 0.01.**(E) **Effect of c-Src on p73 induced Ipaf promoter transctivation. HeLa cells transfected with Ipaf promoter construct along with p73α (2 ng) and c-Src or Hck (100 ng) were subjected to CAT activity measurements. Data presented here are mean ± S.D. of at least three independent experiments, *p < 0.05, **p < 0.01. **(F, G) **Total RNA isolated from Hela cells transfected with HA-p73α (200 ng) or Hck (1300 ng) and mSH3 Hck (1300 ng) were subjected to RT-PCR analysis for Ipaf, MDM2 and PUMA gene expression. p73α and Hck mRNA levels were determined for transfection efficiency control whereas GAPDH mRNA levels were used as an internal control.

Since interacting proteins and modifications affect the transactivation ability of p73, we determined if Hck co-expression influences p73 activity. We first compared the ability of Hck and c-Abl to regulate p73 activity using PG13 construct that has multiple binding sites for p73. c-Abl enhanced p73β activity but Hck co-expression resulted in inhibition of its activity (Fig. 6A). This effect of Hck was also tested using Ipaf promoter. Ipaf, an activator of caspase-1, was recently identified as a p53 target gene induced by DNA damage and was shown to play a role in p53 mediated apoptosis [56]. p73α even at low level (2 ng of plasmid) could strongly transactivate expression of a reporter gene cloned downstream of the Ipaf promoter (Fig. 6B). It was found that Hck repressed activation of Ipaf promoter reporter construct by p73α (Fig. 6B). Coexpression of kinase dead (KD) mutant of Hck was also able to repress p73α-mediated transactivation similar to wild type (WT) Hck when tested using Ipaf reporter construct (Fig. 6B). Overexpression of Hck also repressed p73α-induced transactivation of MDM2 promoter, which was independent of kinase activity of Hck, as co-expression of KD mutant of Hck repressed p73α mediated transactivation similar to WT-Hck (Fig. 6C).

### Functional SH3 domain of Hck is required for inhibition of p73 transcriptional activity

Since Hck inhibited the transcriptional activity of p73α isoform in a kinase-independent manner, we explored the possibility of the involvement of SH3 domain. Towards this end, a critical tryptophan was mutated to alanine (W93A). This mutation in Hck abolished the binding of SH3 domain to proline rich sequences of proteins as well as enhanced its kinase activity [39]. W93A-Hck (mSH3-Hck) was transfected in the presence and absence of p73α along with Ipaf-CAT reporter construct. mSH3-Hck was not able to inhibit p73α mediated transcriptional activity of Ipaf promoter whereas WT-Hck showed inhibition (Fig. 6D), suggesting that this inhibition is dependent on functional SH3 domain of Hck. To analyse if this property was unique to Hck, the effect of Src on Ipaf-promoter transactivation was examined. HeLa cells were transfected with p73α (2 ng) and c-Src or Hck (200 ng each) along with Ipaf-CAT promoter (200 ng) and cell lysates subjected to reporter activity assay. c-Src inhibited p73α mediated transactivation by 26.21% as compared to Hck which inhibited the activity by 60% (Fig. 6E). These results suggest that Hck is a more potent inhibitor of p73 induced Ipaf-promoter transactivation as compared to c-Src indicating the difference in properties between the two members of Src family kinases.

The effect of Hck on p73 induced endogenous gene expression was examined by semi-quantitative reverse transcriptional (RT)-PCR analysis in HeLa cells. Hck and mSH3-Hck were transfected with p73α in a ratio of 6.5:1 and total RNA prepared. These conditions were used to ensure that all p73 expressing cells also co-express Hck. Ectopic expression of p73α resulted in an increase in endogenous Ipaf mRNA levels, and co-expression with WT-Hck resulted in inhibition of expression (Fig. 6F). Co-expression of mSH3-Hck with p73α did not inhibit p73α induced Ipaf mRNA levels (Fig. 6F). This was also observed with endogenous MDM2 mRNA levels where coexpression of WT-Hck inhibited MDM2 gene expression significantly and mSH3-Hck coexpression with p73α did not inhibit p73α induced MDM2 mRNA levels. These results suggested that functional SH3 domain of Hck is indispensable for repression of p73 transcriptional activity. We assessed the ability of Hck to regulate expression of PUMA, a well-known target of p73. RT-PCR analysis showed that p73α expression upregulates expression of PUMA in HeLa cells. Upon co-expression of Hck with p73α, PUMA expression was not altered under the conditions where Ipaf gene expression was repressed (Fig. 6G). This result shows that Hck may regulate endogenous p73 targets selectively.

### Inhibition of p73α induced apoptosis by Hck

**Figure 7:**
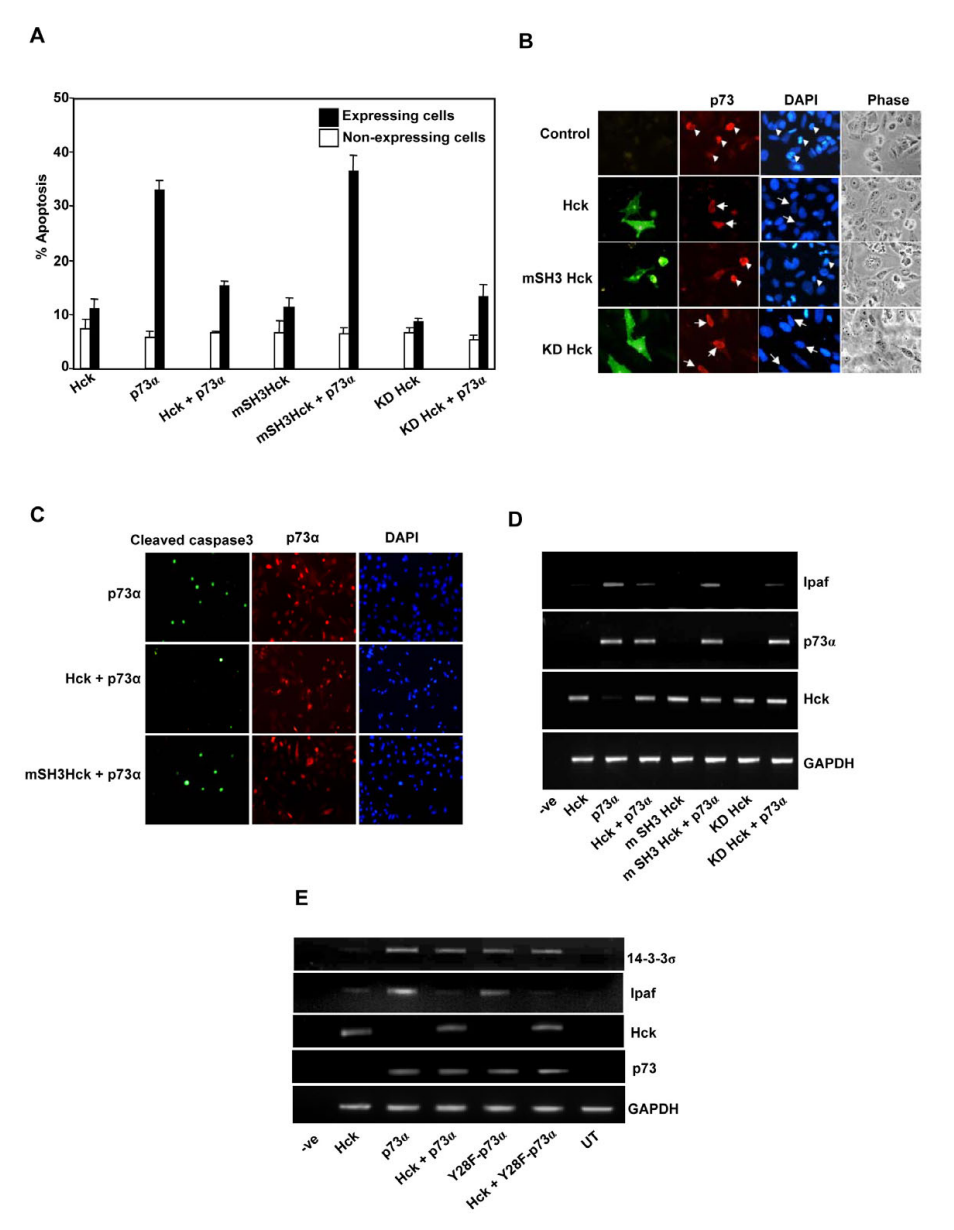
**Hck inhibits p73α-induced apoptosis**. **(A)**. SAOS-2 cells transfected with p73α and Hck, mSH3-Hck or KD-Hck in the ratio of 1:3 were immunostained for p73 and Hck and the percentage of apoptotic cells was scored among expressing and non-expressing cells using morphological criteria. Data represent mean ± S.D. of at least three independent experiments performed on duplicate coverslips. **(B) **Panels show the morphological features of p73 and Hck expressing cells. Arrowhead shows apoptotic cells and arrows indicate healthy cells. **(C) **SAOS-2 cells transfected with p73α and Hck or mSH3-Hck in the ratio of 1:3 were immunostained for p73 and cleaved caspase-3 expression. **(D) **Total RNA isolated from SAOS-2 cells transfected with HA-p73α (200 ng) with or without Hck (1300 ng), mSH3 Hck (1300 ng) and KD Hck (1300 ng) were subjected to RT-PCR analysis as described in Fig. 7E. **(E) **p73α transactivation function is independent of Tyr-28 phosphorylation. RT-PCR analysis was carried out using RNA isolated from SAOS-2 cells after transfection with indicated expression constructs. Expression levels of gene products were determined using appropriate primers. (UT indicates untransfected).

p73α expression induces apoptosis in osteosarcoma cell line SAOS2 that is p53-/- [5]. We wished to determine whether repressive effect of Hck on p73 activity is reflected also in its ability to suppress apoptosis. We examined the effect of Hck expression on p73α-induced apoptosis in SAOS2 cell line. p73α was overexpressed with and without Hck and cells were scored for apoptosis after immunostaining for p73 and Hck expression. p73α alone induced apoptosis in 33% of expressing cells but co-expression with Hck resulted in apoptosis of only 15% of expressing cells (Fig. 7A). A functional SH3 domain in Hck was required for inhibiting p73-induced apoptosis since the W93A-Hck construct did not show any inhibitory effect. This property was independent of Hck kinase activity as seen by the ability of KD Hck to inhibit p73-induced apoptosis (Fig. 7A). The morphology of single and dual expressing cells is shown in Fig. 7B. Further, to assess downstream events of apoptosis, cleaved caspase-3 levels were determined by immunostaining. A large number of p73α expressing cells showed increased staining of cleaved caspase-3, which is a hallmark for apoptosis (Fig. 7C). Co-expression of Hck along with p73α showed significant reduction in number of cells expressing cleaved caspase-3, whereas mSH3-Hck coexpression did not affect the number of cells with cleaved caspase-3 in SAOS2 cells (Fig. 7C).

The effect of Hck on p73 induced Ipaf gene expression in SAOS-2 cells was examined. When Hck was co-expressed with p73α, Ipaf expression was reduced significantly (Fig. 7D). This repression was independent of kinase activity of Hck but dependent on functional SH3 domain of Hck (Fig. 7D). We determined the effect of Y28F-p73α mutation on its ability to transactivate gene expression. Expression of p73α or Y28F-p73α mutant in SAOS2 cells resulted in an increased expression of Ipaf gene as determined by RT-PCR (Fig. 7E). Co-expression of Hck suppressed p73α-induced as well as Y28F mutant-induced expression of Ipaf mRNA (Fig. 7E). To determine whether some other target of p73 is affected by Y28F mutation, we analysed the induction of 14-3-3σ mRNA (known to be induced by p73α) [57] by p73α as well as by Y28F mutant (Fig. 7E). Induction of 14-3-3σ mRNA was seen upon expression of p73α as well as Y28F mutant. Interestingly, there was no suppression of p73α or Y28Fp73α mutant induced 14-3-3σ mRNA by Hck (Fig. 7E). These results suggest that the suppressive effect of Hck on p73-induced gene expression is promoter specific as was also observed in HeLa cells.

**Figure 8:**
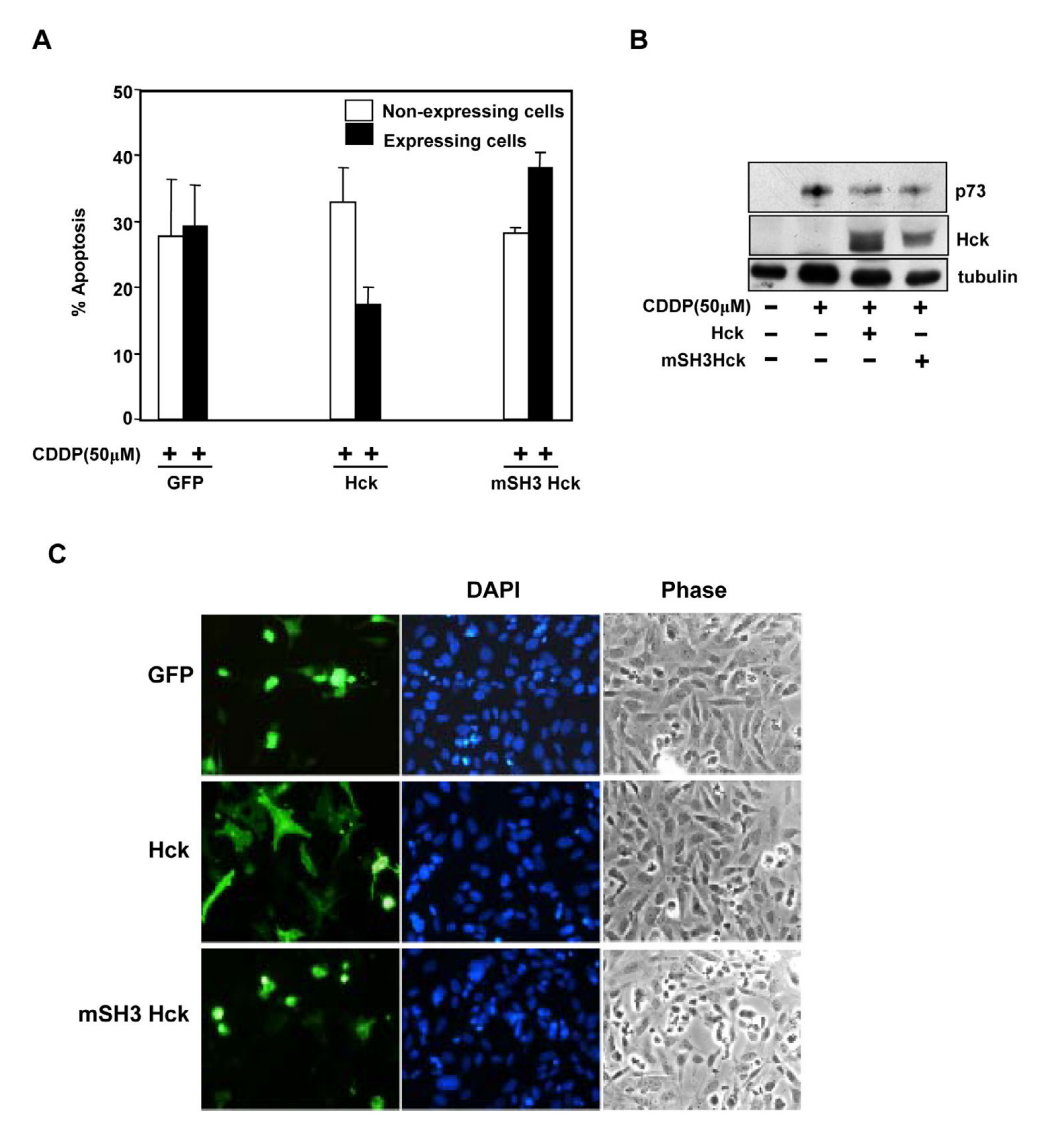
**Hck inhibits cisplatin induced apoptosis**. **(A) **SAOS2 cells transfected with GFP, Hck or mSH3 Hck (400 ng) were treated with 50 μM cisplatin (CDDP) for 24 hours and immunostained for Hck. The percentage of apoptotic cells was scored among expressing and non-expressing cells using morphological criteria. Data represent mean ± S.D. of at least three independent experiments. **(B) **SAOS2 cells transfected with Hck or mSH3 Hck (400 ng) were treated with 50 μM cisplatin (CDDP) for 24 hours and whole cell lysates were subjected to western blotting with p73 (mouse monoclonal, Imgenex) and Hck antibody. Tubulin was used as loading control. **(C) **Panels show the expression of GFP, Hck and mSH3 Hck expressing cells.

Since Hck inhibited p73 induced promoter activation as well as gene expression, this study was also extended to check the ability of Hck to inhibit apoptosis induced by cisplatin, an anti cancer drug known to mediate apoptosis by activation of p73 [12]. SAOS2 cells were transfected with GFP, WT-Hck or mSH3-Hck and immunostained for expressing cells after cisplatin treatment. Quantitation of apoptosis in expressing and nonexpressing cells showed that Hck expression protected 47% of cells from cisplatin induced apoptosis (Fig. 8A). Neither GFP expression nor mutant SH3 Hck expression inhibited this apoptosis. The protein levels of p73α, Hck and mSH3-Hck were also determined upon cisplatin treatment which is shown in Fig. 8B. The morphology of Hck expressing cells is shown in Fig. 8C.

### YAP interacts with Hck and is required for p73-induced activation of Ipaf promoter

**Figure 9:**
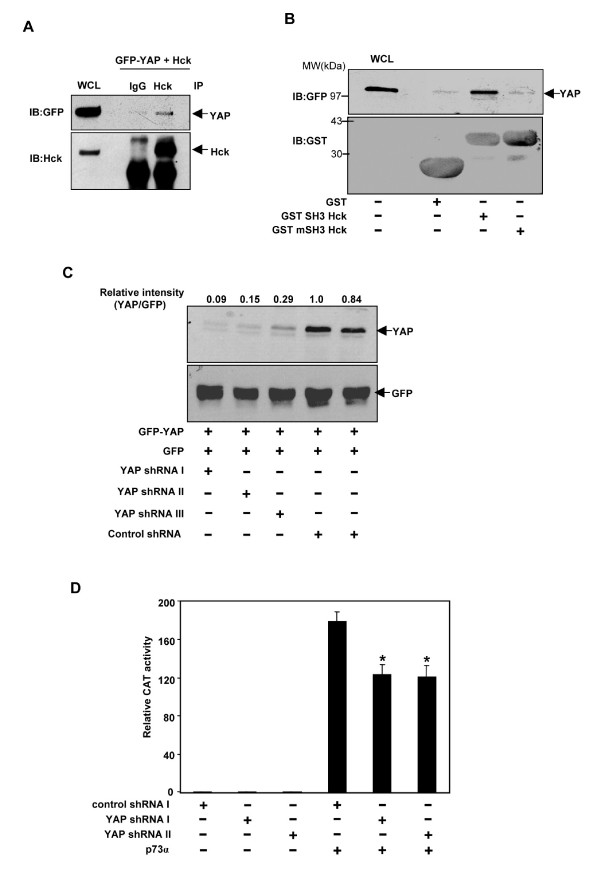
**Hck interacts with YAP and requirement of YAP for transactivation of Ipaf by p73**. **(A) **Cos1 cells transiently expressing GFP-YAP with Hck were immunoprecipitated with control rabbit IgG (C) or Hck polyclonal antibody. The immunoprecipitates were resolved by SDS-PAGE and subjected to western blotting to detect YAP and Hck. **(B) **Extract of Cos1 cells transfected with GFP-YAP was incubated with GST, GST-SH3Hck, GST-mSH3-Hck recombinant proteins bound to Glutathione Sepharose beads. Proteins bound to these beads were immunoblotted with GFP and GST antibodies. **(C) **YAP-shRNA downregulates GFP-YAP expression. Hela cells transfected with GFP-YAP (50 ng) with either control shRNA I and II (200 ng) or YAPshRNA I, II and III (200 ng) subjected to immunoblotting with anti-GFP antibody. GFP (50 ng) was used as transfection efficiency control. **(D)**. Effect of YAP shRNA on p73α mediated Ipaf promoter transactivation. HeLa cells transiently transfected with YAPshRNA I, II (200 ng) or control shRNA I (200 ng) in the presence or absence of p73α (2 ng) along with Ipaf-CAT promoter construct (150 ng) and pCMV-β Gal (50 ng) were subjected to CAT activity measurements.

In an attempt to elucidate the mechanism by which Hck inhibits p73 activity in a target specific manner, we tested the possible involvement of YAP, a molecule that binds to and selectively regulates p73 dependent transactivation [58,59]. YAP was first identified as the Src family kinase Yes, interacting protein [60]. We explored the possibility of YAP interacting with Hck *in vivo *in co-immunoprecipitation experiments. YAP was detected in Hck immunoprecipitates from cells expressing both Hck and YAP and not with control antibody (Fig. 9A). The ability of Hck to interact with YAP was tested *in vitro *using GST fusion proteins. YAP interacted with GST-SH3-Hck domain and not with GST-mSH3-Hck domain (Fig. 9B).

The role of YAP in p73α mediated Ipaf promoter transactivation was determined by knock down of YAP using RNAi strategy. YAP shRNA I, II, III and control shRNA I and II were transfected with GFP-YAP in HeLa cells in the ratio of 4:1 and whole cell lyastes subjected to immunoblotting with GFP antibody. YAP shRNA I, II and III inhibited the expression of GFP-YAP by 90%, 85% and 71%, respectively, as compared to control shRNA I (Fig. 9C). As can be seen in Fig. 9C, (lower panel), transfection efficiency was comparable as determined by GFP blot which was used as an efficiency control. To check the role of endogenous YAP in p73α-induced Ipaf promoter transactivation, YAP shRNA I, II and control shRNA I were transfected with and without p73α along with Ipaf-CAT promoter construct and CAT assays performed after 36 hours of transfection. YAP shRNA I and II significantly inhibited p73α mediated Ipaf-promoter transactivation (P < 0.05) (Fig. 9D). These results suggest that endogenous YAP is required for p73α-induced activation of Ipaf promoter.

## Discussion

The present study identifies p73 as a substrate and interacting partner of the Src family kinase, Hck. *In vitro *studies using a GST fusion protein suggest that p73α could interact directly with SH3 domain of Hck. Hck represses the transcriptional activity of p73α. This repression was observed under conditions where c-Abl co-expression showed enhanced transactivation by p73 indicating that c-Abl and Hck have opposing influence on the function of p73 as a transcriptional activator. This repression was independent of the catalytic activity of Hck but required its SH3 domain. The effect of Hck on p73 activity was also observed at the level of endogenous Ipaf and MDM2 gene expression but did not influence two other known targets of p73, PUMA and 14-3-3σ. The selective regulation of p73 targets by Hck suggests a distinct role for Hck *in vivo *with respect to modulation of expression of certain genes. Apoptosis induced by exogenously expressed, as well as endogenous p73, was inhibited by Hck, dependent on its SH3 domain protein interaction function. This property of Hck may therefore be a consequence of its ability to repress target gene induction by p73.

Hck mediated inhibition of p73 activity may be effected by direct interaction between these two proteins *in vivo*, or by the involvement of a Hck interacting protein that is required for p73 dependent transcriptional activation. Endogenous as well as exogenously expressed Hck has earlier been shown to localize to plasma membrane, Golgi, secretory granules, nongranular membranes and cytosol [47-50]. Most intracellular functions of Src family kinases have been attributed to their localization to the plasma membranes and cytosolic compartments. More recently tyrosine kinases involved in receptor mediated signaling pathways have been shown to be present in the nucleus and their role in influencing activity of transcription factors has been described [54,61]. Our results suggest that both exogenously expressed as well as endogenous Hck is present to some extent in nuclear compartment of cells. These observations raise the possibility that the inhibition of p73 transcriptional activity by Hck is mediated at least in part by the fraction of Hck present in the nucleus. Cytoplasmic sequestration of p73 by Hck is not likely to explain inhibition of p73 activity by Hck because the level of p73 in the nucleus (which is likely to be the transcriptionally active component) does not decrease upon co-expression of Hck. Hck interaction with p73 directly could alter its ability to bind or transactivate a set of targets selectively.

Our results also show that Hck interacts with YAP, a transcriptional co-activator, which imparts target selectivity to p73. Since YAP was required for maximal activation of Ipaf promoter by p73, it is possible that Hck mediates repression of p73 transcriptional activity through its ability to interact with YAP. Based on the ability of p73δ to interact with Hck and not with YAP [62], we can infer that Hck and YAP bind to different sequences on p73. Formation of multimolecular complex, whereby both Hck and YAP interact with p73, could be responsible for Hck causing selective repression of p73 target genes. Since Hck can bind to YAP or to p73 through its single SH3 domain, an alternate possibility is that its interaction with YAP may alter the ability of YAP to transactivate p73 targets.

Phosphorylation of p73 by c-Abl at Y99 results in its activation [23]. Our study shows that Hck predominantly targets a tyrosine in the transactivation domain (Y28). This difference in target site beween Hck and c-Abl rules out the possibility of Hck expression resulting in p73 phosphorylation through activation of c-Abl, although Hck is known to activate c-Abl [42]. This modification (Y28 phosphorylation) did not influence the activity of p73 because Y28F mutant showed the same level of activation of Ipaf promoter as WT p73. Hck inhibited Ipaf gene expression induced by p73α as well as Y28F mutant. We also demonstrated that this modification is not a unique property of Hck, since c-Src expression also resulted in predominant phosphorylation of p73 at Y28. In addition, Hck also targets other tyrosine residues (which we have not specifically identified) because Y28 mutant was not totally deficient in phosphorylation.

p73 stabilization has generally been shown to reflect in an increase in its activity [12-14,24]. Our study showed that over- expression of Hck results in stabilization of p73 protein, dependent on its kinase activity, but this does not lead to an increase in p73 activity. Similarly, it has been reported earlier that interaction of p73 with MDM2 results in p73 stabilization but inhibition of p73 transcriptional activity [63]. Our results also show that activation of endogenous Hck results in tyrosine phosphorylation of p73. p73 protein levels increase upon differentiation of mylemonocytic cells ([43] and present study). Under these conditions of differentiation, Hck levels and activity are also known to increase. Hck activity mediated signaling may therefore contribute to enhanced protein levels of endogenous p73 upon differentiation. Upon cell fractionation, we observed that Hck co-expression enhanced p73 protein levels only in the cytosolic fraction, but not in the nuclear fraction. Phosphorylated p73 was essentially seen in the cytosolic fraction. p73 localized to nuclear and cytoplasmic compartments may have independent cellular functions just as does p53 [64-66]. Since phosphorylation at Y28 by Hck does not appear to play a significant role in its stability, it is likely that other mechanisms contribute to p73 stabilization dependent on the kinase activity of Hck. Recently, Fyn, a Src family kinase was shown to negatively regulate Itch by tyrosine phosphorylation, impairing its ubiquitinating activity [67]. Since Itch regulates cellular p73 levels by causing its degradation [68], it is also possible that SFKs may indirectly regulate p73 by preventing its degradation. We have yet to determine the role of p73 stabilized by the kinase activity of Hck in the cytoplasmic compartment.

SFKs modulate diverse signaling cascades dependent on their ability to interact with and phosphorylate various target proteins. In some contexts kinase and adaptor functions can act independently to effect downstream signaling. It therefore, appears that stabilization, which is catalytic activity dependent and suppression of apoptosis, which is SH3 domain interaction dependent, are independent effects of Hck on p73. The functional significance of Y28 phosphorylation of p73 is not clear at present and would require further investigation.

## Conclusion

In conclusion, our results show that the tyrosine kinase Hck interacts with p73α physically and functionally. Hck and c-Src phosphorylate p73α at Tyr-28, a novel site of phosphorylation located in the transcriptional activation domain. Transcriptional activity of p73 towards certain target genes is selectively inhibited by Hck independent of its kinase activity. Hck-SH3 domain mediated interactions play an important role in the inhibition of p73-dependent transcriptional activation of target genes as well as apoptosis.

## Methods

### Cell culture and transfections

HeLa, Cos1, and human osteosarcoma SAOS-2 cells were grown in Dulbecco's modified eagle medium supplemented with 10% fetal bovine serum and antibiotics. HL-60 cells were maintained in RPMI 1640 medium with heat- inactivated 10% fetal bovine serum and antibiotics. Cultures were maintained in a humidified 37°C incubator with 5% CO_2_. Transient transfections of HeLa and SAOS-2 were performed with LipofectAMINE Plus reagent according to the manufacturer's recommendations (Invitrogen). For transfection of Cos1 cells cationic lipid DHDEAB was used as described [50].

### Plasmid constructs and antibodies

The mammalian expression plasmids used were: hemagglutinin (HA) epitope-tagged p73α or p73β in pcDNA3 (gift from Dr. Gerry Melino, University of Rome, Italy), Human p59 Hck in pcDNA6 (gift from Dr. Todd Miller, State University of New York, Stony Brook), c-Src and K297R-c-Src (from Upstate Biotechnology, Lake Placid, New York), GFP-YAP (gift from Dr. Marius Sudol, Mount Sinai School of Medicine, New York), GST-p73α (gift from Dr. Giovanni Blandino, Regina Elena Cancer Institute, Italy), p53/p73 responsive promoter construct PG13-Luc (kindly provided by Dr. Bert Vogelstein, Johns Hopkins University, Baltimore), MDM2-Luc promoter construct (kind gift from Dr. Moshe Oren, Weizmann Institute of Science, Israel), and Ipaf-CAT (pCAT-P2) promoter construct, described by us earlier [56]. Different p73α mutants (tyrosine to phenyl alanine) were made based on tyrosine phosphorylation sites having high scores using the NetPhos 2.0 program for SFK target sites prediction. Y28F-p73α, Y99F-p73α, Y121F-p73α, Y309F-p73α, Y355-56F-p73α, and Hck mutants, K269E-Hck, W93A-Hck were made by PCR based site directed mutagenesis. Plasmids expressing GST-SH3 and GST-mSH3 domain of Hck (amino acids 72-143) were prepared by cloning the appropriate PCR product into BamH1 and EcoR1 sites of pGEX2T. All the sequences of the constructs were confirmed by using an automated DNA sequencer. The green fluorescence protein (GFP) expression plasmid pEGFP-C1 was from Clontech. Antibodies to p73 (H-79, rabbit ployclonal), Hck (rabbit polyclonal), PY20 (mouse monoclonal), α-tubulin (mouse monoclonal), c-Src (goat polyclonal), Calnexin (rabbit polyclonal) and GST (mouse monoclonal) were purchased from SantaCruz Biotechnology. p73 monoclonal antibody clone 1288 was from Imgenex. PARP antibody (rabbit polyclonal) was from Roche.

### Construction of vector expressing YAP shRNA

The YAP shRNA expression vector was constructed using the U6 promoter-based vector essentially as described [56,71]. The YAP sequence targeted by shRNA (GenBank™ accession NM_006106) was from nucleotides 792-812 (for YPI), 1570-1590 (for YPII) and 1254-1274 (for YPIII). The YAP sequences targeted by shRNAs were a) YPI: 5'-GACATCTTCTCGTCAGAGATA-3' b) YPII: 5'-GCTGCCACCATGCTAGATAAA-3' and c) YPIII: 5'-CCTTAACAGTCGCACCTATCA-3'. The vectors expressing shRNA of unrelated sequence of the same length were used as control. All the sequences were confirmed by automated DNA sequencing.

### Immunoprecipitation, GST pull down and Western blot analysis

Cos1 cells transfected with indicated plasmids were lysed in lysis buffer (50 mM Tris-Cl pH 7.5, 150 mM NaCl, 10% glycerol, 0.5%NP-40, 2 mM EDTA, 2 mM EGTA, 2 mM PMSF, 2 mM NaF, 2 mM Na_3_VO_4 _and protease inhibitor (Roche Biochemicals) and the extract was incubated with antibodies overnight at 4°C. The immune complexes were captured using Protein A/G Plus beads (Santa Cruz) and washed with buffer (50 mM Tris-Cl pH7.5, 150 mM Nacl, 10% Glycerol, 0.1% NP-40), the proteins were eluted by boiling in 3× SDS-sample buffer and separated on 8% SDS PAGE followed by western blotting with the required antibodies using ECL detection reagent (Perkin Elmer).

For GST pull down assays, cultures of E. Coli DH5α expressing GST, GST-SH3Hck or GST-mSH3-Hck were induced by 1 mM isopropyl-β-D-thiogalactopyranosidase (IPTG) for 4 hours at 37°C. Cells were lysed by addition of cold PBS containing 1 mM PMSF and protease inhibitors (Roche) and sonicated. To this, 1% Triton-X 100 was added for 30 minutes at 4°C for solubilization and then centrifuged to remove insoluble materials. To the supernatant, Glutathione Sepharose beads (50% slurry) were added and incubated with end-to-end shaking on Rototorque at 4°C for 1 hour. Beads were pelleted, washed with PBS containing 0.1% TritonX-100 and incubated 6–8 hours with lysates of Cos1 cells transiently transfected with indicated plasmids. Bound proteins were eluted by boiling in 3× SDS sample buffer and subjected to immunoblotting.

### *In-vitro *phosphorylation assay

Purified recombinant human Hck (kind gift from Dr. John Kuriyan, UC Berkeley) was activated for 30 minutes at 37°C in 20 μl kinase buffer (10 mM TRIS-Cl pH 7.5, 0.5 mM DTT, 10 mM MgCl_2_, 1 mM MnCl_2_) containing 20 μM Na_3_VO_4 _and protease inhibitors. Activated Hck (80 nM) was incubated with GST and GST-p73α in the presence of 3 μCi of γ^32^P-ATP for 30 minutes at 37°C. The reaction was terminated by the addition of 3X-SDS sample buffer followed by boiling for 5 minutes. The proteins were analyzed by 10% SDS-PAGE and gel was stained with commassie blue to visualize expression of proteins and subsequently dried for phosphor image analysis.

### Treatment of cells with mercuric chloride

HL-60 cells were grown in RPMI medium with 10% heat-inactivated fetal calf serum. These cells were differentiated by the addition of DMSO (1.25%) for 48 hours. Differentiated cells were subjected to HgCl_2 _treatment as described by Robbins *et al*. [46]. Briefly, the cells were washed with phosphate-buffered saline and treated with 0.5 mM HgCl_2 _for 15 minutes at room temperature before the lysates were subjected to immunoprecipitation with p73 (rabbit polyclonal) antibody.

### Cell fractionation

Transfected Cos1 and HL-60 cells were suspended in cold buffer A (10 mM Hepes-KOH pH7.9, 1.5 mM MgCl_2_, 10 mM KCl, 1 mM DTT, 1 mM PMSF, 2 mM Na_3_V0_4_, 1 mM EDTA, 1 mM EGTA, protease inhibitors) and incubated on ice for 15 minutes. Later, NP-40 was added to 1% v/v, vortexed for 20 seconds and incubated on ice for 10 minutes. Cells were pelleted down at 1500 g for 5 minutes at 4°C. Supernatant, which is the cytosolic extract, was re-suspended in 3× SDS sample buffer and boiled for 5 minutes. The nuclear pellet was washed thrice with cold buffer A (without NP-40) and re-suspended in 3× SDS sample buffer, boiled for 5 minutes and resolved by 8% SDS PAGE and subsequently immunoblotted with the required antibodies.

### Apoptosis assays and Immunofluorescence staining

Quantitative analysis of apoptotic cells was carried out as described previously [36,69]. Cells grown on coverslips were transfected and processed for immunostaining using appropriate antibodies for detection of expressing cells. Cells were mounted in 90% glycerol containing 1 mg/ml para-phenylenediamine (antifade) and 0.5 μg/ml DAPI (4'6-diamidino-2 phenylindole) for DNA staining. Cells showing immunofluorescence staining were counted and those cells that showed loss of refraction, condensed chromatin, apoptotic bodies, cell shrinkage were scored as apoptotic. At least 200 expressing cells were counted in each coverslip. The data represent the mean ± S.D. from at least three independent experiments on duplicate coverslips. Background apoptosis was determined by counting non-expressing cells in the same coverslips. For immunofluorescence staining of endogenous Hck protein, HL-60 cells grown on coverslips were fixed after differentiation for 24 hours with 10 ng/ml of 12-*O*-tetradecanoylphorbol-13-acetate (TPA) and stained with Hck (rabbit polyclonal) primary antibody overnight at 4°C, followed by anti-rabbit Cy3 secondary antibody incubation for 45 minutes.

### Reporter assays

Hela cells were seeded in 24 well plates and transfected with indicated promoter constructs and expression plasmids along with pCMV.SPORT-βGal (Life technologies, Inc.) Total DNA was kept constant to 400 ng by the use of pcDNA3. Cells were lysed in reporter lysis buffer (Promega, Corp.) after 30 hours of transfection and luciferase activity measured by Luciferase repoter assay system (Promega, Corp). CAT assay was carried out as described previously [70]. Relative luciferase or CAT activities were calculated after normalizing with β-galactosidase enzyme activities.

### RT-PCR Analysis

HeLa and SAOS2 cells were transfected with indicated combination of expression plasmids and after 30 hours of transfection, total RNA was isolated using Trizol reagent (Life Technologies, Inc.). Semi-quantitative PCR was carried out as described previously [70]. Appropriate primers for Ipaf, MDM2, PUMA, 14-3-3σ, Hck, p73 and GAPDH were used for amplification.

## Authors' contributions

All experiments were carried out by PP. VR helped with apoptosis assays. The project was conceived by GS and VR. Design of experiments and interpretation of data was done by all three authors. All authors contributed to writing of the paper and approved the final manuscript.
